# Sport-Specific Assessment of the Effectiveness of Neuromuscular Training in Young Athletes

**DOI:** 10.3389/fphys.2018.00264

**Published:** 2018-04-11

**Authors:** Erika Zemková, Dušan Hamar

**Affiliations:** ^1^Department of Sports Kinanthropology, Faculty of Physical Education and Sport, Comenius University in Bratislava, Bratislava, Slovakia; ^2^Sports Technology Institute, Faculty of Electrical Engineering and Information Technology, Slovak University of Technology in Bratislava, Bratislava, Slovakia

**Keywords:** agility, balance, core stability, muscle strength and power, speed, testing

## Abstract

Neuromuscular training in young athletes improves performance and decreases the risk of injuries during sports activities. These effects are primarily ascribed to the enhancement of muscle strength and power but also balance, speed and agility. However, most studies have failed to demonstrate significant improvement in these abilities. This is probably due to the fact that traditional tests do not reflect training methods (e.g., plyometric training vs. isometric or isokinetic strength testing, dynamic balance training vs. static balance testing). The protocols utilized in laboratories only partially fulfill the current needs for testing under sport-specific conditions. Moreover, laboratory testing usually requires skilled staff and a well equipped and costly infrastructure. Nevertheless, experience demonstrates that high-technology and expensive testing is not the only way to proceed. A number of physical fitness field tests are available today. However, the low reliability and limited number of parameters retrieved from simple equipment used also limit their application in competitive sports. Thus, there is a need to develop and validate a functional assessment platform based on portable computerized systems. Variables obtained should be directly linked to specific features of particular sports and capture their complexity. This is essential for revealing weak and strong components of athlete performance and design of individually-tailored exercise programs. Therefore, identifying the drawbacks associated with the assessment of athlete performance under sport-specific conditions would provide a basis for the formation of an innovative approach to their long-term systematic testing. This study aims (i) to review the testing methods used for the evaluation of the effect of neuromuscular training on sport-specific performance in young athletes, (ii) to introduce stages within the Sport Longlife Diagnostic Model, and (iii) to propose future research in this topic. Analysis of the literature identified gaps in the current standard testing methods in terms of their low sensitivity in discriminating between athletes of varied ages and performance levels, insufficent tailoring to athlete performance level and individual needs, a lack of specificity to the requirements of particular sports and also in revealing the effect of training. In order to partly fill in these gaps, the Sport Longlife Diagnostic Model was proposed.

## Introduction

All of us are aware of the importance of reaching the correct diagnosis to identify the illness and craft a prompt treatment. Though sport cannot be compared with clinics, the right and accurate assessment of athlete performance is crucial for designing effective training programs. Getting the right diagnostics is also a key for physical fitness development in general population. It provides useful information on their strengths and weakness and in this way it may reveal potential health risks. By focusing exercise programs on these aspects, it may prevent some type of diseases or injuries before they ever occur.

So far, most of the diseases have been linked to the elderly population; however today, more than ever before they affect middle-aged people (e.g., low back pain), and even the young generation (e.g., diabetes). This issue could be related to workplace conditions (occupational stress, manual operations, etc.) and/or a sedentary way of life. Identifying symptoms associated with a particular disease by assessing the body balance, muscle strength and power or hamstring flexibility may be considered as a “preventive” tool before it starts to become a chronic disease.

Although several field tests have been proposed for various populations, most of them do not meet the requirements of the modern age. One of their main shortcomings is the insufficient reliability and limited number of variables due to the very simple equipment used. Therefore, there is a need to apply portable computerized systems for testing both athletes and untrained subjects of different ages and fitness levels. Only on the basis of relevant variables, their performance capabilities may be evaluated and appropriate exercise programs designed. Testing batteries should include user-friendly, portable and low-cost diagnostic systems well suited for the testing of varied groups in a relatively short time period. These batteries might be applicable in clinical settings, as well as in non-medical institutions, fitness centers or school sports clubs.

In particular, testing batteries for young individuals should get a makeover. So far, a variety of them have been developed for school age children. In Europe, it is the Eurofit Physical Fitness Test Battery, devised by the Council of Europe, that has been used in many European schools since 1988. This test battery includes nine tests assessing speed, strength, endurance and flexibility. Despite the many advantages of field tests, these do not sufficiently reflect various aspects of physical fitness relevant to a particular age and are not sensitive enough to exercise induced changes specific to a particular sport. These traditional methods of assessing the physical fitness of children and adolescents only partially fulfill current needs for testing under sport-specific conditions.

Preliminary analysis of the literature identified the gap in current standard testing methods in terms of their low sensitivity in discriminating between young athletes of varied ages and performance levels and specificity to reveal the effect of training. This study aims (i) to review the existing testing methods used for the evaluation of the effect of neuromuscular training on sport-specific performance in young athletes, (ii) to introduce stages within the Sport Longlife Diagnostic Model, and (iii) to propose future research in this topic.

## Methods

Two specific questions were addressed in this review: (1) Can existing testing methods effectively evaluate changes in sport-specific performance of young athletes following neuromuscular training? and (2) Which of the current tests are suitable to be implemented in the Sport Longlife Diagnostic Model?

In order to answer these questions and to reveal the gaps in the current literature, we provided a literature search. Electronically available literature was searched using the MEDLINE database, PubMed, SportDiscus® and Web of Science. Additional searches were performed on SpringerLink, Elsevier, EBSCOhost and Google Scholar. Besides peer-reviewed journal articles, available conference proceedings were analyzed. Search results were limited to studies closely related to the main topic of this review—to identify methods used for assessment of the effectiveness of neuromuscular training on physical fitness in children and adolescents. Our primary focus was on testing under sport-specific conditions. However, this approach resulted in the identification of a small number of studies that were considered eligible for the review. Therefore other relevant studies that evaluated the effect of training on at least one measure of neuromuscular functions and/or athletic performance were included. This help us to identify the gap in current testing methods using for evaluation of changes in neuromuscular performance after specific training in a particular sport.

The target population was young competitive athletes coupled with other groups of children and adolescents involved in physical activities in sport clubs or schools. The most frequent terms “neuromuscular training,” “resistance training,” “strength and power training,” “plyometric training,” “muscular fitness,” “muscle strength,” “muscle power,” and “muscular endurance” used for research procedure were combined with particular sports (basketball, gymnastics, soccer, swimming, tennis, volleyball, etc.). Further searches were conducted using relevant words from each subheading, namely tests used for assessment of muscular fitness. The key inclusion criterion was that studies evaluated the effects of neuromuscular training on sport-specific athlete performance. In consequence of limited research in this field, studies investigating the effects of such a training on traditional measures of muscle strength and power, core stability and strength, body balance, agility and speed were also included. These abilities represent a crucial aspect of performance in many sports, and therefore their assessment should be an integral part of testing in young athletes. Studies were excluded if tests or testing batteries were not sufficiently specified. Studies that failed to meet these conditions were excluded from this review.

## Results and discussion

### Overview of test batteries for assessment of physical fitness in children and adolescents

Fitness testing is a common part of the curriculum in many schools. These testing programs vary across regions, countries and continents.

One of the first were the American Alliance for Health, Physical Education, Recreation and Dance (AAHPERD) test battery, developed in 1957, which included pull-up, standing long jump, flexed leg sit-up, shuttle run, 50-yard dash, and 600-yard run (option: 1 mile or 9 min run for 10–12 years old, and 1.5 miles or 12 min run for ≥13 years old) (AAHPERD, 1976), and the Canadian Association for Health, Physical Education and Recreation (CAHPER) test battery, which dates back to 1963, and included flexed arm hang, standing long jump, 1 min speed sit-ups, shuttle run, 50 m run and endurance run (800 m run for 6–9 years old, 1,600 m run for 10–12 years old, and 2,400 m run for 13–17 years old) (CAHPER, 1980).

In 1994, AAHPERD adopted FITNESSGRAM as its national fitness testing program. The FITNESSGRAM Health-related fitness test battery consists of following tests: bent arm hang (included in 1987), pull up (included in 1987, removed in 2005), 90° push-up (included in 1992), modified pull up (included in 1992), curl-up (included in 1992), trunk lift (included in 1992), modified sit-up test (included in 1987, removed in 1992), one mile run/walk (included in 1987), one mile walk test (included in 1999), PACER test—a 20 m progressive, multi-stage shuttle run (included in 1992), shuttle run K-3 (included in 1987, removed in 1992), sit and reach test (included in 1987, removed in 1992), shoulder stretch (included in 1992), and back saver sit and reach (included in 1992; Plowman et al., 2006).

However, in September 2012 the New Presidential Youth Fitness Program was launched. This program is focused on assessing health rather than the athleticism of America's youth. The updated version was intended to assess health related fitness of youth with emphasis on their personal goals. This new program was developed in partnership with experts in youth fitness and health promotion including the Amateur Athletic Union, the American Alliance for Health, Physical Education, Recreation and Dance, the Centers for Disease Control and Prevention and the Cooper Institute.

In Europe, the most used is the Eurofit Physical Fitness Test Battery (Council of Europe, Committee for the Development of Sport, 1993) that includes plate tapping—tests speed of limb movement, handgrip test—measures static arm strength, bent arm hang—measures muscular endurance/functional strength, standing broad jump—measures explosive leg power, sit-ups in 30 s—measures trunk strength, 10 × 5 m shuttle run—measures running speed and agility, 20 m endurance shuttle run—tests cardiorespiratory endurance, flamingo balance test—single leg balance test, and sit-and-reach—flexibility test. Another example is the Assessing Levels of Physical Activity (ALPHA) health-related fitness test battery for children and adolescents. This includes a 20 m shuttle run test, handgrip strength test, standing broad jump, and a 4 × 10 m shuttle run test (Ruiz et al., 2011).

Further examples are: International Physical Fitness Test (United States Sports Academic, General Organization of Youth and Sport of Bahrain), Amateur Athletic Union Test Battery (Chrysler Foundation, Amateur Athletic Union), YMCA Youth Fitness Test, National Youth Physical Program (the United States Marines Youth Foundation), Fit-4-Fun test battery, Canadian Physical Activity, Fitness and Lifestyle Approach (Canadian Society for Exercise Physiology), National Fitness Test Program in the Popular Republic China (China's National Sport and Physical Education Committee), Australian Fitness Education Award (the Australian Council for Health, Education and Recreation), New Zealand Fitness Test (Rusell, Department of Education), and so forth.

In general, these batteries include tests assessing endurance (e.g., 20 m shuttle run, Cooper test–12 min run, 9 min run, cycle ergometer sub-maximal test, 1.5 mile run/walk test, 1 mile run/walk test, 1/2 mile run/walk test, 1/4 mile run/walk test, 1,000 m run), speed and agility (e.g., 50 m sprint or 50 yard run, 100 m dash, shuttle run for 4 × 10 m or 10 × 5 m, shuttle run with sponges for 10 × 4 m, plate tapping), muscle strength (e.g., handgrip, medicine ball or basketball throw, bent arm hang, push-ups and their modifications, standing broad jump, vertical jump—countermovement jump, Abalakov jump and Sargent jump—vertical jump tests with the arms swing, sit-ups and their modifications–7-stage, 30, 60 s, or up to failure, trunk lift), balance (e.g., Flamingo balance test), and flexibility (e.g., sit and reach, stand and reach, V sit and reach, and shoulder stretch).

Similar test batteries consisting primarily of strength tests have been used to evaluate the effect of neuromuscular training on physical fitness in children and adolescents (Table 1). These batteries include mainly repetition maximum (1, 6, 10 RM) strength test on various exercises (e.g., bench press, squat, leg press, knee extension, elbow flexion), isometric strength tests (e.g., leg extensors on a leg press), medicine ball throwing, push-ups, curl-ups, handgrip, repeated leg press or chest press exercise, standing long jump, vertical jump (e.g., squat jump, countermovement jump, drop jump, jumping sideways, triple hop, single-leg hop, maximal and submaximal hopping), Bourban trunk muscle strength test—assesses core strength endurance, speed and agility tests (sprints at 10, 20, or 30 m, shuttle run, Illinois agility test), PACER test—multistage, 20 m shuttle run, half mile run, one mile run, or other cardiorespiratory fitness tests, static and dynamic balance tests (e.g., standing on a stable platform or those exposed to perturbations, Stork stand balance test, Y balance test, Star excursion balance test), flexibility tests (stand and reach, sit and reach, V sit and reach, shoulder stretch), and other more specific tests based for instance, on coordination tasks.

**Table 1:** Overview of tests for assessment of the effect of neuromuscular training on physical fitness of children and adolescents.

**Authors, year**	**Type of training**	**Duration (weeks)**	**Subjects**	**N (E/C)**	**Sex**	**Age (years)**	**Tests**
Alves et al., 2016	Strength/Strength and aerobic	8	Schoolchildren	41, 45, 38/44	F,M	10–11	Medicine ball throwing (1 and 3 kg) Standing long jump Countermovement jump 20 m sprint
Andrejić, 2012	Strength/Plyometric and strength	6	Basketball players	10+11	M	12–13	Vertical jump Long jump Seated medicine ball toss 20 m sprint 4 × 15 m standing start running Stand and reach flexibility test
Annesi et al., 2005	Cardiovascular, resistance, flexibility	12	Schoolchildren	570	F,M	5–12	Push-ups Shoulder stretch 1 mile run/walk
Assunção et al., 2017	Plyometric	10	Track and field athletes, volleyball, basketball, and soccer players	14/15		~16.79, 16.85	30 m sprint 1600 m aerobic test Running anaerobic sprint test
Barber-Westin et al., 2010	Neuromuscular	6	Tennis players	15	F,M	~13.0	Single-leg hop for distance test Single-leg triple crossover hop for distance test Baseline speed and agility forehand and backhand tests Service box speed and agility tests Single-court and 2-court suicide runs Abdominal endurance test
Behringer et al., 2013	Resistance/Plyometric	8	Tennis players	12, 12/12	M	~15.03	10 repetition maximum test Service velocity test Service precision test
Benson et al., 2007	Resistance	8	Intermediate and high-school students	37/41		10–15	1 repetition maximum bench press and leg press Cardiorespiratory fitness test
Bishop et al., 2009	Plyometric	8	Swimmers	11/11		~13.1, 12.6	Swimming block start test
Brown et al., 1986	Plyometric	12	High school basketball players	13/13	M	~15.0	Vertical jump without arms swing Vertical jump with arms swing
Buchheit et al., 2010a	Explosive strength/Repeated shuttle sprint	10	Soccer players	7, 8	M	~14.5	10 and 30 m sprints Repeated shuttle sprint ability test Countermovement jump Hopping test
Buchheit et al., 2010b	Speed-agility/Sprint interval	4	Handball players	7, 7	M	~16.0, 16.0	Countermovement jump 10 m sprint Repeated sprint ability test Graded intermittent aerobic test (30–15 Intermittent Fitness Test)
Cavaco et al., 2014	Strength and plyometric	6	Soccer players	5, 5/6	M	~13.8, 14.2/14.2	1 repetition maximum squat 15 m sprint 15 m agility test with the ball Crossing efficiency and shooting efficiency tests
Channell and Barfield, 2008	Olympic/Traditional resistance	8	High school athletes	11, 10/6	M	~15.9	Vertical jump 1 repetition maximum squat and power clean
Chaouachi et al., 2014	Plyometric/Balance and plyometric	8	Schoolchildren	14, 14/12	M	12–15	1 repetition maximum leg press Horizontal and vertical jumps Triple hop for distance test Reactive strength test Leg stiffness test 10 and 30 m sprints Standing Stork test Star excursion balance test Shuttle run test
Chaouachi et al., 2017	Balance and plyometric	8	Soccer players	26	M	~13.9	Isometric back and knee extension strength tests Countermovement jump Standing long jump Triple hop test Maximal and submaximal hopping test 10 and 30 m sprints 49 m shuttle run test Standing Stork test Y-balance test
Chelly et al., 2009	Strength	8	Soccer players	11/11	M	~17.0	Cycling force-velocity test Squat jump Countermovement jump 5-jump test 40 m sprint 1 repetition maximum back half squat at 90 degrees
Chelly et al., 2014	Plyometric	8	Handball players	12/11	M	~17.4	Force-velocity ergometer tests for upper and lower limbs Squat jump Countermovement jump Video filming of sprint velocities (first step, first 5 m, and 25–30 m)
Chelly et al., 2015	Plyometric	10	Track athletes	14/13	M	~11.9	Cycle ergometer force-velocity test Squat jump Countermovement jump Drop jump Multiple 5-bound test 40 m sprint
Christou et al., 2006	Soocer, strength and soccer	16	Soccer players	9, 9/8	M	12–15	1 repetition maximum leg press and bench press Squat jump Countermovement jump Repeated jumps for 30 s 30 m sprint 10 × 5 m shuttle run Seat and reach flexibility test Soccer technique
Coutts et al., 2004	Resistance	12	Rugby players	21/21	M	~16.7	3 repetition maximum bench press and squat Maximal chin-ups Countermovement jump with the arm swing 10 and 20 m sprints
da Fontoura et al., 2004	Strength	12	Schoolchildren	7/7	M	~9.4/9.7	1 repetition maximum knee extension and elbow flexion
Diallo et al., 2001	Plyometric	10	Soccer players	10/10	M	12–13	Countermovement jump Squat jump Drop jump Multiple 5-bound test Repeated rebound jumps for 15 s 20, 30, and 40 m sprints Force-velocity cycling test
Drinkwater et al., 2005	Bench press	6	Basketball and soccer players	12+14	M	~18.6, 17.4	6 repetition maximum bench press Smith machine bench throw with 40 kg
Escamilla et al., 2010	Baseball conditioning	4	Baseball players	17/17		11–15	Throwing velocity test
Faigenbaum et al., 1993	Strength	8	Schoolchildren	15/10	F,M	8–12	10 repetition maximum strength test Sit and reach flexibility test Vertical jump Seated ball put
Faigenbaum et al., 1996	Strength	8	Schoolchildren	15/9	F,M	7–12	6 repetition maximum leg extension and chest press Vertical jump Sit and reach flexibility test
Faigenbaum et al., 1999	Resistance	8	Schoolchildren	15, 16/12	F,M	5.2–11.8	1 repetition maximum strength and muscular endurance tests on the leg extension and chest press
Faigenbaum et al., 2001	Resistance	8	Schoolchildren	15, 16, 12, 11/12	F,M	5.2–11.8	1 repetition maximum vertical chest press Local muscular endurance test on the vertical chest press
Faigenbaum et al., 2002	Strength	8	Schoolchildren	21+34	F,M	7.1–12.3	1 repetition maximum chest press and leg press Handgrip strength test Long jump Vertical jump Sit and reach test
Faigenbaum et al., 2005	Resistance	8	Schoolchildren	23+20	F,M	8.0–12.3	1 repetition maximum chest press Local muscular endurance test (15 RM) on the leg press Long jump Vertical jump V-sit flexibility test
Faigenbaum and Mediate, 2006	Medicine ball	6	High-school students	69/49	F,M	15–16	Shuttle run test Long jump Sit and reach flexibility test Abdominal curls Medicine ball push-up Medicine ball seated toss
Faigenbaum et al., 2007a	Plyometric and resistance/Resistance	6	Baseball and American football players	13, 14	M	12–15	Vertical jump Long jump Seated medicine ball toss 9.1 m (10 yd) sprint Pro-agility shuttle run test V-sit flexibility test
Faigenbaum et al., 2007b	Resistance	9	Schoolchildren	22	M	~13.9	10 repetition maximum squat and bench press Vertical jump Seated medicine ball toss Sit and reach test PACER test (multistage, 20 m shuttle run)
Faigenbaum et al., 2009	Plyometric	9	Schoolchildren	40/34	F,M	8–11	Standing long jump Sit and reach flexibility test Curl-up test Push-up test Shuttle run test Half mile run
Faigenbaum et al., 2013	Muscular fitness	8	Schoolchildren	20/19	F,M	~7.5/7.6	Standing long jump Single-leg hop Curl-up test Stork stand balance test
Falk and Mor, 1996	Resistance and martial arts	12	Schoolchildren	14/15	M	6–8	Sit-ups for 20 s Seated ball put Standing broad jump Sit and reach flexibility test 6 × 4 m shuttle run Coordination task
Fernandez-Fernandez et al., 2013	Strength	6	Tennis players	15/15	M	13	Service velocity test Service accuracy test Shoulder internal/external rotation test
Ferrete et al., 2014	Strength and high-intensity	26	Soccer players	11/13		8–9	15 m sprint Countermovement jump Yo-Yo intermittent endurance test Sit and reach flexibility test
Filipa et al., 2010	Neuromuscular	8	Soccer players	13/7	F	~15.4/14.7	Star excursion balance test
Flanagan et al., 2002	Strength	11	Third-grade students	14, 24/20	F,M	~8.75, 8.64, 8.65	Two-handed medicine ball put Standing long jump Shuttle run test
Gabbett et al., 2008	Strength, conditioning, and skills	10	Rugby players	14+21		~14.1, 16.9	Vertical jump 10, 20, and 40 m sprints 505 Agility test Multistage fitness test
González-Badillo et al., 2005	Resistance	10	Lifters	16, 17, 18	M	~16.4, 16.5, 16.8	1 repetition maximum snatch, clean and jerk, and back squat
González-Badillo et al., 2006	Heavy resistance	10	Weightlifters	12, 9, 8	M	~17.1, 16.9, 17.5	1 repetition maximum snatch, clean and jerk, and back squat
Gorostiaga et al., 1999	Heavy resistance	6	Handball players	9, 10/4	M	14–16	Maximal isometric knee extensions and flexions 1 repetition maximum leg press and pec-dec Squat jump Countermovement jump Throwing a handball ball Running test
Gorostiaga et al., 2004	Strength	11	Soccer players	8/11	M	~17.2	Countermovement jump 5 and 15 m sprints Endurance running test
Granacher et al., 2011	Ballistic strength	8	High-school students	14/14		~16.7, 16.8	Isometric strength test of the leg extensors on the leg press Countermovement jump One-legged balance test Mediolateral perturbation test
Granacher et al., 2014	Core strength (stable, unstable)	6	Physically active adolescents	13, 14	F,M	13–15	Bourban TMS test Standing long jump 20 m sprint Stand and reach test Jumping sideways test Emery balance test Y balance test
Granacher et al., 2015	Plyometric (stable, highly unstable)	8	Soccer players	12, 12	M	~15.2, 15.6	Countermovement jump Drop jump Multiple 5-bound test 30 m linear sprint Figure-8 run agility test One-legged balance test Star excursion balance test Mediolateral perturbation test
Hammami et al., 2016	Balance and plyometric	8	Soccer players	24	M	12–13	Medicine ball throw Countermovement jump Maximal and submaximal hopping test 4 × 9 m shuttle run 10, 20, and 30 m sprints Standing Stork balance test Y-Balance test
Häkkinen et al., 1989	Endurance, sprint, strength	1y	Endurance runners, sprinters, weightlifters	4, 4, 4+6/6	M	11–13, 17/11–12	Multistage treadmill run test Isometric strength of the leg extensor muscles Countermovement jump Maximal squat lift
Keiner et al., 2014	Strength	2y	Soccer players	62/50		13–18	Change of direction sprint test 1 repetition maximum/body mass in the squat
King and Cipriani, 2010	Plyometric	6	High school basketball players	16, 16		14–16	Vertical jump
Klusemann et al., 2012	Supervised or video-based resistance	6	Basketball players	13, 13/12	F,M	14–15	20 m sprint Step-in vertical jump Agility test Sit and reach test Line drill test Yo-Yo intermittent recovery test (level 1) 15-s push-ups and pull-ups Functional movement screening
Kotzamanidis et al., 2005	Combined high-intensity strength and speed	9	Soccer players	12, 11/12	M	~17.0, 17.1, 17.8	Squat jump Countermovement jump Drop jump from the height of 40 cm 30 m dash 1 repetition maximum for each exercise
Kotzamanidis, 2006	Plyometric	10	School students	15/15		~11.1/10.9	Squat jump Running at distances 0–10, 10–20, 20–30, and 0–30 m
Lehnert et al., 2009	Plyometric	8	Volleyball players	11	F	~14.8	Standing vertical jump Vertical jump with an approach Shuttle run for 6 × 6 m
Lehnert et al., 2012	High resistance/Plyometric	5	Soccer players	8/8		~17.8/17.8	Isokinetic strength of knee flexors and extensors Countermovement jump 10 and 30 m sprints
Lehnert et al., 2017	Pre-season conditioning (jumping, strength)	8	Volleyball players	12	F	~16.8	Countermovement jump with free arms Squat jump with hands on the hips Drop jump with hands on the hips Isokinetic knee flexion and extension test
Lephart et al., 2005	Plyometric and resistance	8	Athletes	14, 13	F	~14.5, 14.2	Isokinetic knee flexion and extension torque test Isometric hip abduction torque test Drop-landing test
Lloyd et al., 2012	Plyometric	4	School students	41, 44, 44	M	9, 12, 15	Maximal and submaximal hopping test
Maio Alves et al., 2010	Strength	6	Soccer players	9, 8/6	M	~17.4	Squat jump Countermovement jump 5 and 15 m sprints 505 Agility test
Martel et al., 2005	Aquatic plyometric	6	Volleyball players	19	F	~15.0	Vertical jump Knee extension and flexion strength test
Matavulj et al., 2001	Plyometric	6	Basketball players	11, 11/11	M	15–16	Countermovement jump Isometric tests of hip and knee extensors
Meylan and Malatesta, 2009	Plyometric	8	Soccer players	14/11	M	~13.3, 13.1	10 m sprint Agility test Squat jump Countermovement jump Contact test Multiple 5-bound test
Michailidis et al., 2013	Plyometric	12	Soccer players	24/21	M	~10.6, 10.6	Graded exercise test on a treadmill 30 m sprint with a 10 m splits Standing long jump Multiple 5-bound test Squat jump Countermovement jump Drop jump from the height of 30 cm 10 repetition maximum barbell squat 30 s Wingate test Soccer-specific tests (agility, kicking distance)
Muehlbauer et al., 2012	High-velocity strength	8	High-school students	14/14	F,M	16–17	Isometric strength test on the leg press Countermovement jump
Mujika et al., 2009	Sprint and power	7	Soccer players	10, 10	M	~18.1, 18.5	Countermovement jump without and with arms swing 15 s countermovement jumps 15 m sprint 15 m agility test
Myer et al., 2005	Neuromuscular	6	Basketball, soccer and volleyball players	41/12	F	13–17	Countermovement jump 9.1 m sprint Single-leg hop and hold distance test 1 repetition maximum squat and bench press Three-dimensional biomechanical analysis
Negra et al., 2016	Resistance and soccer/Soccer	12	Soccer players	13, 11		~12.8, 12.7	1 repetition maximum half-squat Countermovement jump Squat jump Standing long jump Multiple 5-bound test 5, 10, 20, and 30 m sprints *T*-test Illinois change of direction speed test
Negra et al., 2017	Resistance/Plyometric	12	Soccer players	12, 11/11		~12.8	20 m sprint Illinois change of direction speed test Countermovement jump Squat jump Standing long jump Multiple 5-bound test 1 repetition maximum half-squat
Nikolaidis, 2015	Plyometric	10	Soccer players	11/10	M	~11.4/11.3	Standing long jump 10 and 30 m sprints
Potdevin et al., 2011	Plyometric	6	Swimmers	12/11	F,M	~14.3, 14.1	Squat jump Countermovement jump Swim tests (a gliding task, 400 and 50 m front crawl with a diving start) 2 tests with a water start without push-off on the wall (25 m in front crawl and 25 m only with kicks)
Prieske et al., 2016	Core strength (stable, unstable)	9	Soccer players	20, 19	M	~17.0	Tests of trunk muscle strength/activation Countermovement jump 20 m linear sprint T-agility test Kicking performance test
Ramírez-Campillo et al., 2013	Plyometric	7	High school students	9, 8, 7/5	M	~16.89	Strength tests (5 maximum repetitions) Drop jumps from heights of 20, 40, and 60 cm Squat jump Countermovement jump Timed 20 m sprint Illinois agility test
Ramirez-Campillo et al., 2014a	Plyometric	7	Soccer players	13, 14, 12/15	M	~10.4	Countermovement jump Drop jump from heights of 20 and 40 cm 20 m sprint Change of direction speed test Kicking performance test
Ramírez-Campillo et al., 2014b	Low-volume high-intensity plyometric	7	Soccer players	38/38	M	~13.2, 13.2	20 m sprint Illinois agility test Countermovement jump Drop jump from heights of 20 and 40 cm Multiple 5-bound test Maximal kicking test for distance 2.4 km time trial
Ramírez-Campillo et al., 2015a	Vertical, horizontal, and combined vertical and horizontal plyometric	6	Soccer players	10, 10, 10/10	M	10–14	Vertical and horizontal countermovement jumps with arms Multiple 5-bound test Drop jump from the height of 20 cm Maximal kicking velocity test Sprint test Change of direction speed test Yo-Yo intermittent recovery test (level 1) Balance tests (normal stance, eyes open; normal stance, eyes closed)
Ramírez-Campillo et al., 2015b	Plyometric	6	Soccer players	8, 8/8	M	~13.0	Bilateral and unilateral horizontal and vertical countermovement jumps with arms Drop jump from the height of 20 cm Maximal kicking velocity test 10 m sprint Change of direction speed test Yo-Yo intermittent recovery test (level 1)
Ramírez-Campillo et al., 2015c	Plyometric	6	Soccer players	54, 57/55	M	10–17	Squat jump Countermovement jump Drop jump from the height of 20 cm Broad long jump 20 m sprint 10 × 5 m agility test 20 m multistage shuttle run test Sit and reach test
Ramírez-Campillo et al., 2015d	Unilateral, bilateral, and combined plyometric	6	Soccer players	16, 12, 12/14	M	10–15	Unilateral and bilateral countermovement jumps with arms Multiple 5-bound test Drop jump from the height of 20 cm Maximal kicking velocity test 15 and 30 m sprints Agility test Yo-Yo intermittent recovery test (level 1) Bilateral balance tests (normal stance, eyes open; normal stance, eyes closed; perturbed stance, eyes open; perturbed stance, eyes closed)
Rhea et al., 2008	Plyometric	12	High school athletes	32, 32	F,M	~17.4	Countermovement jump
Roden et al., 2014	Strength (squats, CMJs)	6	High school basketball players	20	M	~15.4	Countermovement jump
Rubley et al., 2011	Plyometric	14	Soccer players	10/6	F	~13.4	Vertical jump Kicking distance test
Saeterbakken et al., 2011	Core stability	6	High-school handball players	14/10	F	~16.6	Throwing velocity test
Sáez de Villarreal et al., 2015	Plyometric and sprint	9	Soccer players	13/13		14–15	10 m sprint 10 m agility test with and without the ball Countermovement jump Abalakov vertical jump Yo-Yo intermittent endurance test Ball-shooting speed test
Sander et al., 2013	Strength	24	Soccer players	134		U13,U15,U17	1 repetition maximum front and back squat 30 m linear sprint
Sankey et al., 2008	Plyometric	6	Rugby players	6, 6/6	M	~14.5	Countermovement jump Drop jump
Santos and Janeira, 2008	Weight and plyometric	10	Basketball players	15/10	M	14–15	Squat jump Countermovement jump Abalakov test Depth jump Mechanical power test Medicine ball throw
Santos and Janeira, 2009	Resistance and plyometric	10	Basketball players	8, 7	M	14–15	Squat jump Countermovement jump Abalakov test Depth jump from a 40 cm platform Mechanical power test Seated medicine ball throw
Santos and Janeira, 2011	Plyometric	10	Basketball players	14/10	M	~15.0, 14.5	Squat jump Countermovement jump Abalakov test Depth jump Mechanical power test Medicine ball throw
Santos and Janeira, 2012	Resistance	10	Basketball players	15/10	M	14–15	Squat jump Countermovement jump Abalakov test Drop jump from a 40 cm platform Seated 3 kg medicine ball throw
Siegler et al., 2003	Plyometric, resistance, and high-intensity anaerobic	10	High school soccer players	17/17	F	~16.5, 16.3	Abridged 45 min shuttle test Vertical jump 20 m running-start sprint 30 s Wingate test
Soh et al., 2007	Aerobic and strength	8	Sport school netball players	21		14–18	SEMO Agility run test Vertical jump
Söhnlein et al., 2014	Plyometric	16	Soccer players	12/11		11.2–14.7	20 and 30 m sprints Hurdle agility run 5 × 10 m shuttle run Multiple 5-bound test Standing long jump
Szymanski et al., 2007a	Resistance plus medicine ball	12	High school baseball players	25/24	M	~15.4	3 repetition maximum torso rotations at the dominant and nondominant side Sequential hip-torso-arm rotational strength test (medicine ball hitter's throw) 3 repetition maximum parallel squat and bench press
Szymanski et al., 2007b	Resistance plus medicine ball	12	High school baseball players	25/24	M	~15.4	3 repetition maximum torso rotations at the dominant and nondominant side Sequential hip-torso-arm rotational strength test (medicine ball hitter's throw) 3 repetition maximum parallel squat and bench press
Thomas et al., 2009	Plyometric	6	Soccer players	15	M	~17.3	Countermovement jump 20 m sprint with a 5 m splits 505 agility test
Thompson et al., 2017	Resistance	6	High school athletes	38	M	15–18	1 repetition maximum back squat Countermovement jump
Tsimahidis et al., 2010	Heavy resistance combined with running	10	Basketball players	13/13		~18.0/18.0	1 repetition maximum half squat 10 and 30 m sprints Squat jump Countermovement jump Drop jump
Tsolakis et al., 2004	Resistance	8	Untrained preadolescents	9/10	M	11–13	Isometric strength test 10 repetition maximum elbow flexion
Weston et al., 2015	Isolated core	12	Swimmers	10/10	F,M	~15.7, 16.7	50 m front-crawl swim Straight-arm latissimus dorsi pull-down test Timed prone-bridge test Analysis of EMG activity of the core muscles while performing MVCs
Wong et al., 2010	Strength and power	12	Soccer players	28/23	M	~13.5/13.2	Countermovement jump Ball-shooting test 30 m sprint Yo-Yo intermittent endurance run (level 1) VO_2_max test
Zribi et al., 2014	Plyometric	9	Basketball players	25/26	M	~12.1/12.2	30 m sprint Squat jump Countermovement jump Countermovement jump with arms swing 5-jump test Force-velocity test on the cycle ergometer

### Overview of tests for assessment of the effect of neuromuscular training on athlete performance

Analysis of the literature identified (Table 1) that the efficiency of neuromuscular training (namely resistance and plyometric or in combination with balance, agility and other exercises) was evaluated mainly by repetition maximum tests (1, 3, 6, 10 RM) for different exercises (bench press, squat, leg press, power clean, snatch, clean & jerk, torso rotations, etc.), isokinetic and isometric tests (back, knee extension and flexion), force-velocity test on a cycle ergometer, 30 s Wingate test, standing long and vertical jumps (e.g., squat jump, countermovement jump, Abalakov test, drop jump, multiple 5 bounds test, repeated rebound jumps, triple hop test, maximal and submaximal hopping), standing and seated medicine ball throw, 15 s push-up or pull-up, agility tests (e.g., 505 Agility test, T-agility test, Illinois agility test, hurdle agility run, shuttle run for various distances, i.e., 6 × 6 m, 4 × 9 m, 4 × 15 m, 10 × 5 m) and/or change of direction speed tests, sprints at different distances (5, 10, 15, 20, 30, and 40 m), balance tests (e.g., bilateral stance with eyes open and eyes closed, perturbed stance with eyes open and eyes closed, Standing stork balance test, Y-balance test, Star excursion balance test, etc.), endurance tests (e.g., Yo-Yo intermittent endurance test, Yo-Yo intermittent recovery test, 20 m multistage shuttle run test, multistage running test on a treadmill), and flexibility tests (e.g., stand and reach, sit and reach, V sit and reach).

However, only few sport-specific tests were used for this purpose. These include throwing a handball ball, 10 or 15 m agility test with the ball, service velocity and accuracy tests in tennis, single-court and 2-court suicide runs in tennis, kicking velocity test in soccer, crossing efficiency and shooting efficiency tests in soccer, swim tests (a gliding task, 400- and 50 m front crawl with a diving start), tests with a water start without push-off on the wall (25 m in front crawl and 25 m only with kicks), swimming block start performance test, and so forth.

These findings indicate that moving from traditional field tests to more sophisticated testing methods evaluating athletic performance under sport-specific conditions would be a key step forward. So far, physical fitness of children and adolescents has been assessed using simple equipments (no PC-based), although various portable computerized diagnostic devices are available on the market. Therefore current test batteries should be updated. These novel batteries should be able to objectively assess athletic abilities and skills by means of novel technologies and computational techniques using for data analysis over a long-term period. This should be supported by web-based access to standard and sport-specific test protocols, management of data obtained, and their reporting. This would be the first fundamental step in proposing objective measurement tools that use technological advances in the physical fitness testing of young athletes.

### Long-term sport diagnostic model

The first long-term athlete development (LTAD) model was proposed in Canada in 1998 by Dr. Istvan Balyi and was grounded around three phases: Training to Train, Training to Compete, and Training to Win. Over time, this model was evolved into seven phases: (1) Active Start, (2) FUNdamentals, (3) Learn to Train, (4) Train to Train, (5) Train to Compete, (6) Train to Win, and (7) Active for Life (Balyi et al., 2013).

Recently, Granacher et al. (2016) presented a conceptual model for the implementation of resistance training programs during the stages of long-term athlete development to enhance muscular fitness and athletic performance. According to the authors, long-term development of muscular fitness (strength, power, and endurance) consists of these stages: “early childhood (female: 6–8 years, male: 6–9 years) including coordination training, agility training, balance training, muscular endurance training with own body mass/training tools (e.g., medicine ball) with a focus on exercise technique; late childhood (female: 9–11 years, male: 10–13 years) including balance training, plyometric training as part of deliberate play (e.g., rope skipping) with a focus on correct jumping and landing mechanics, core strength training, muscular endurance training with own body mass/training tools (e.g., medicine ball), free weight training with a focus on exercise technique; adolescents (female: 12–18 years, male: 14–18 years) including balance training, plyometric training (depth jumps from low drop heights), core strength training, free weight training at light to moderate loads, heavy resistance strength training (hypertrophy), eccentric resistance training, sport-specific resistance training; and adulthood (female: >18 years, male: >18 years) including balance training, plyometric training (depth jumps from moderate drop heights), core strength training, free weight training at moderate to high loads, heavy resistance strength training (neuromuscular activation + hypertrophy), sport-specific resistance training.”

Taking this information into account, our previously proposed Long-Term Sport Diagnostic Model (Zemková, 2015) was modified. Age-related stages within this model are as follows: Stage 1 (6–9 years), Stage 2 (10–14 years), Stage 3 (15–18 years), Stage 4 (19–24 years), Stage 5 (25–44 years), Stage 6 (45–64 years), and Stage 7 (65+ years).

Targeting the young population, we have originally developed tests specifically tailored for them. So far, reliable and sensitive parameters were identified that are directly linked to the physical fitness of particular age categories and allow capture of the complexity of the performance by combining multiple parameters. Their combination showed superior results for the accurate assessment of different abilities when compared to current standard field tests. Experience showed that young people participate more intensively and also reach higher exercise goals than with conventional methods when computerized diagnostic and training systems are used. For instance, the task-oriented balance tests based on visual feedback control of body position or the agility test performed under simulated competitive conditions seem to be more suitable for children and adolescents than traditional field tests. Both of them are similar to computerized games which may enhance the attention and motivation of children to exercise. We believe that this approach may also be applied for testing of young athletes under sport-specific conditions.

Besides basic tests of reaction, agility and speed, core and postural stability, muscle strength and power, the Spinal Mouse® device was also used to assess the spinal curvature and pelvic tilt. We have tested 118 children and adolescents for sit-and-reach, passive and active straight leg raise (Muyor et al., 2014). The findings identified that the sit-and-reach test is an appropriate and valid test for the evaluation of the pelvic tilt and lumbar flexion, but not as a measure of hamstring flexibility in school age children. The active straight leg raise test may be an appropriate and easy test for the assessment of their hamstring flexibility. It may be recommended also for young athletes as most of the authors used flexibility tests in their studies evaluating the efficiency of neuromuscular training.

**Figure 1:**
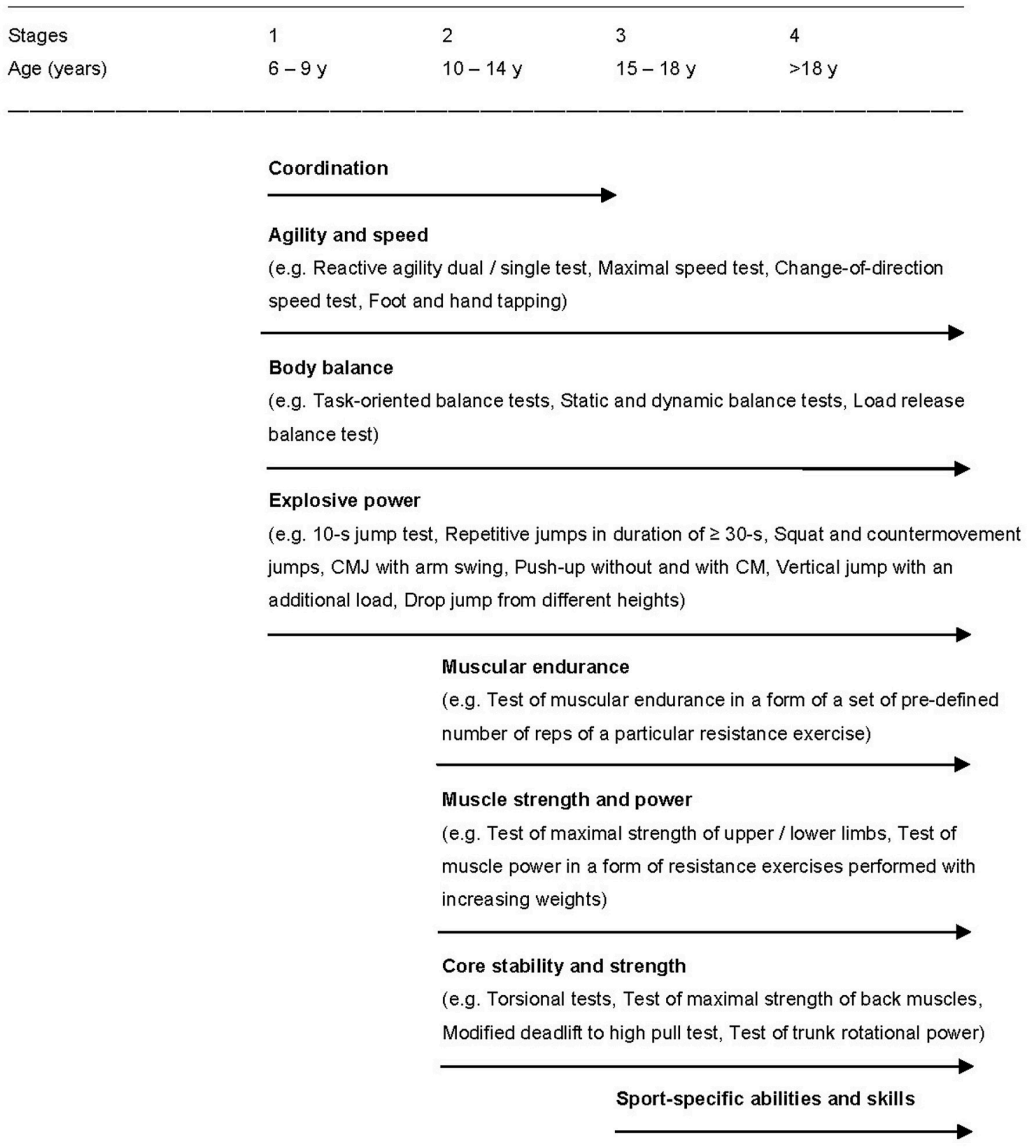
Proposed tests within the abridged Long-Term Sport Diagnostic Model for evaluation of the effect of neuromuscular training on physical fitness in young athletes.

The focus in the present study was given to testing of neuromuscular functions in children and adolescents (Figure 1). Proposed tests can be adjusted according to requirements of particular sports and serve as a basis for Sport-Specific Model of Athlete's Performance Testing. The motive for research in this field was our experience with several years of systematic testing of athletes, for instance, those of the National Karate Team (aged from 9 to 27 years). The most used tests were as follows: reaction test (e.g., responses to stimuli of red and blue during the strike of gyaku-cuki), hand and foot tapping, kicking velocity test, agility test, 10 and 60 s jump tests, 10 s exercise bouts at different revolution rates on the isokinetic cycle ergometer, a 30 s load on the isokinetic cycle ergometer or on a treadmill in the form of tethered running, and spiroergometry (Zemková and Dzurenková, 2004). Though these tests were found to be suitable for assessment of performance in karate competitors, prescribed testing protocols used in laboratories only partially fullfil the requirements for testing under sport-specific conditions. Obtaining relevant data on changes in sport-specific athlete performance during the long-term period would provide more useful information for the designing of effective training programs.

#### Sport-specific assessment of muscle strength and power in young athletes

The ability to produce power during running, hopping and jumping improves as children grow older. The age-related increase in power production can mainly be ascribed to the increase in muscle size (Kanehisa et al., 1994; Neu et al., 2002). However, Davies and Young (1984) and Ferretti et al. (1994) showed that differences in muscle mass cannot fully explain differences in peak power between pre-adolescent children and adults. According to Ferretti et al. (1994) age-related differences in neural drive could play a role. However, Lambertz et al. (2003) found that the stiffness of the musculotendinous unit increases throughout childhood. This suggests that stiffness of lower limbs may contribute to developmental changes in jump performance (Wang et al., 2004). These findings were extended by Korff et al. (2009) who reported a significant correlation between peak power during the countermovement (CM) jump and lower limb stiffness in adolescents but not in pre-adolescents. When normalized to body mass, the relationship between peak power and stiffness of lower limbs differed similarly between these groups. These findings indicate that leg stiffness may contribute to greater power production during jumps in adolescents. On the other hand, the ability to produce power during vertical jumps in pre-adolescents is not related to the leg stiffness. This weak relationship may be explained by a greater compliance of passive elastic structures in pre-adolescents (Asai and Aoki, 1996; Lambertz et al., 2003). Alternatively, a lesser ability to actively stiffen their joints by antagonistic co-activation (Hortobagyi and DeVita, 2000) may result in lower ability of intersegmental control (Jensen et al., 1994). Taking into account that active (Arampatzis et al., 2001) as well as passive (Bobbert, 2001) stiffness components influence jump performance, pre-adolescents have a lesser ability to actively stiffen their joints to produce power (Korff et al., 2009). This may be strengthened by the greater compliance of their passive elastic structures (Lambertz et al., 2003). They can benefit from elastic energy storage in the musculotendinous system during CM jumps (Korff et al., 2009).

Therefore, the estimation of the ability to utilize elastic energy across maturational stages could provide useful information on the long-term development of jump performance and reveal potential for its trainability. From the exercise physiology it is known that activation of the stretch-shortening cycle during CM exercise contributes to greater power than the one performed without a prior eccentric contraction (Bosco et al., 1982). Thus, the greater jump height difference between the countermovement and the squat jump indicates an enhanced ability to utilize the elastic energy. Similar parameter can be obtained during resistance exercises performed with and without CM. Using an additional load during jumps or squats enables better differentiation of power performance in highly skilled athletes.

However, such an enhancement of power due to CM may differ between jumps and squats, depending on demands on the utilization of elastic energy during athlete performance (Zemková et al., 2017c). While this potentiating effect is greater during jumps than squats in high jumpers and volleyball players, opposite is true for rock & roll competitors in acrobatics and dancing. More specifically, the values are significantly higher during squats than jumps in acrobatic rock & roll competitors, whereas there are no differences in those who dance. On the other hand, the values are significantly higher during jumps than squats in indoor volleyball players, whereas there are no differences in those performing on the sand. Differences in CM potentiation of power in the concentric phase of jumps may also be observed in volleyball players playing different positions (spikers, blockers and setters). However, this enhancement of power during jumps and squats does not differ significantly either in hockey players or in karate competitors. These differences between groups of athletes may be ascribed to specific adaptations to exercise modes used during plyometric and resistance training.

There is a number of variations of plyometric training, including repetitive jumps on and off a box and jumping while wearing weight belts (Bobbert et al., 1996). To determine the height at which the highest power is achieved, one has to perform drop jumps, in random order, from different heights. Then, the relationship between jump height and/or power output and drop height can be described. Alternatively, the optimal drop jump height for plyometric training can be estimated.

During drop jumps, the reactive strength index (RSI), which represents a ratio of jump height and ground contact time (McClymont, 2003), is usually calculated. However, this parameter can also be calculated from repetitive jumps. There is a moderate correlation between RSI calculated from drop jump and repetitive jumps (*r* = 0.67). This method of calculating RSI from repetitive jumps may be used for athletes performing rebound jumps (e.g., aerobic & rock and roll dancers) because it is closer to their specific demands than drop jump.

For instance, appropriate selected tests can reveal the different characteristics of jumping between male and female rock & roll dancers. Boys' jump height is significantly higher when jumps are performed with bent knees than with straight legs. However, jump height does not differ significantly between these test conditions in girls. They achieve significantly higher power in the concentric phase of take off than boys. These differences may be attributed to similar muscle work during jumps in the test and rock & roll performance. While girls perform bounces mainly with legs straight, boys used to jump with knees bent.

Training can lead to specific changes in power production during repetitive hopping in dancers with different forms of muscle activity during rhythmic movements. Rock & roll dancers produce significantly higher power in the concentric phase of take-off than aerobic dancers and synchronized swimmers, concomitant with a significantly lower ground contact time. For them, the beneficial effects of an increased recoil speed from stiffer muscle-tendon units might outweigh the increased energy cost at higher jumps and contribute to lower fatigue index during such an exercise.

The fatigue index represents a decline of power during repeated vertical jumps, usually in a duration of 30, 60, or 90 s, depending on sport specialization. Assessment of muscular endurance is of special interest in sports like aerobic gymnastics or rock & roll, where explosive strength should be maintained for the prescribed period of the performance.

In other sports, shorter 10 s test of maximal jumps can be used to assess the explosive power of lower limbs. It can also be utilized for indirect estimation of muscle fiber distribution in lower limbs because there is a high correlation between the percentage of fast twitch muscle fibers in the vastus lateralis and power produced during 15 s jump test (*r* = 0.86) (Bosco et al., 1983). Such an information can be useful for talent identification.

As can be seen from this analysis, various approaches have been used to assess jump performance, however little is known about their advantages and limitations, particularly when testing children. Their great variability in jumping, a lack of familiarization with proper technique of the jump, or their potential learning effects in a short period of time might influence jump performance and consequently its changes across maturational stages. Therefore, there is a need to design a monitoring tool that would reveal various aspects of jump performance in the particular age, provide reliable data and be sensitive to developmental changes specifically in girls and boys.

Besides plyometric training, most of the authors in the studies analyzed used resistance exercises for the improvement of neuromuscular performance in young athletes. Estimating maximal power using the maximal effort single repetitions with increasing weights is considered as a more suitable alternative for the assessment of strength capabilities in adolescents than traditional 1 RM approach. Subjects usually perform the exercise with stepwise increasing weights up to maximal power. However, one has to be aware that maximal values of peak power and mean power in the acceleration phase of resistance exercises are achieved at lower weights than maximal values of mean power produced over the whole concentric phase, for instance at ~50 and 60% 1 RM respectively during bench presses and at ~70 and 80% 1 RM respectively during squats (Zemková et al., 2014).

Currently, instability resistance exercises are often a part of athletic and health-oriented strength training programs (Zemková, 2016c). Therefore, their role in sport-specific performance and general physical fitness is a matter of interest among conditioning specialists and researchers. We have found that measurement of peak and mean power during chest presses on a Swiss ball provides reliable data comparable to those obtained during bench presses under all conditions tested (Zemková et al., 2015a) and may represent an appropriate method for evaluation of the effects of instability resistance training. However, peak values of power measured during chest presses on an unstable surface with weights ≥80% 1 RM should be interpreted with caution.

Besides chest presses and squats, muscle power can be evaluated via many other resistance exercises with free weights or using weight stack machines (e.g., leg press). Some examples are knee extensions and knee flexions. Mean power measured during these exercises is a reliable and also a sensitive parameter discriminating groups with different levels of physical activity (Zemková et al., 2015b). It can also be used for assessing the differences between the injured and non-injured leg. In addition, muscular endurance of knee extensors and knee flexors can be evaluated using the fatigue index calculated from a set of repetitions (i.e., 15). Such an assessment of muscle power during resistance exercises should be implemented in the functional diagnostics of young athletes and so complement existing testing methods.

#### Sport-specific assessment of core stability and strength in young athletes

The importance of the function of the core for body stabilization and force generation in many sports is being recognized. The “core” is described as a box with the abdominals in the front, paraspinals and gluteals in the back, the diaphragm as the roof, and the pelvic floor and hip girdle musculature as the bottom (Richardson et al., 1999). Core strength involves the strength of trunk muscles, whereas core stability reflects the control of trunk position and its motion over the pelvis and leg in order to allow force production to the terminal segment in integrated kinetic chain exercises (Kibler et al., 2006).

Measurement of core stability involves the incorporation of variables of balance and coordination. The majority of core stability tests require the individual to keep a neutral spine in a quadrupedal or supine position (Liemohn et al., 2005; Faries and Greenwood, 2007; Gamble, 2007) that involves activation of local core muscles, such as the transversus abdominus and multifidus. Other tests assess the static muscular endurance of global core muscles, for example external obliques, quadratus lumborum and erector spinae (McGill, 2002; McGill et al., 2003; Faries and Greenwood, 2007). The most used are the Biering-Sørensen test of lumbar extension (Biering-Sørensen, 1984) and the flexor and side bridge endurance tests (McGill, 2001) which are exclusively performed isometrically, usually to task failure.

Another example are instrumented torsional tests, which can be performed under stable or unstable conditions. The task of the subject is to take a correct push-up position with hands on the dynamometric platform while legs are supported on the bench or physioball. Another alternative is that the subject gets into the back bridge position with legs on the dynamometric platform and back supported on the bench or physioball. These tests can also be performed in more difficult conditions with either one hand or one leg placed on the dynamometric platform. During these tests, basic stabilographic parameters are registered using the posturography system based on the dynamometric platform.

Field testing of core strength involves the amount of weight lifted, the number of repetitions performed, and the time of maintenance of neutral stable position (Faries and Greenwood, 2007). In the laboratory, isometric and isokinetic dynamometers are frequently used. In the sporting field, the back dynamometer or a potrable version of the computer-based device allowing the measurement of maximal voluntary isometric strength of predominantly back muscles can be used.

Given that muscle power is a better indicator of athletic performance, the test that measures this parameter during trunk movement may be more specific. The exercise in a form of deadlift to high pull may best mimic the demands imposed by sports comprising of lifting tasks. Muscle power measured during this exercise with free weights and on the Smith machine is a reliable and sensitive parameter able to distinguish the lifting performance in healthy young individuals (Zemková et al., 2016a).

Implements, such as the medicine ball and cable pulleys that allow motion in all three planes, can also be very useful in testing of strength and power performance. Variables of both medicine ball throws and the chop and lift have shown high reliability (Kohmura et al., 2008; Palmer and Uhl, 2011; Rivilla-Garcia et al., 2011; Lehman et al., 2013). Similarly, Andre et al. (2012) reported that a pulley trainer system and an external dynamometer represent a reliable tool for assessing the power during trunk rotations in a sitting position. Such a test may be appropriate for canoeing or kayaking, however for other sports, such as hockey or tennis, standing trunk rotations would be a more relevant alternative. The test adapted from the standing cable wood chop exercise on a weight stack machine is a reliable to assess the maximal power and endurance of core muscles and sensitive to differences among physically active individuals (Zemková et al., 2017a).

Such a computer-based system that can be directly connected to the weights on a stack machine is applicable for testing in fitness centers. Though machines are good for training or testing of muscle strength and power, they neglect key stabilization components of the core. Using free weights is a way to “functional” training and testing because it places greater demands on stabilizing muscles and allows a full range of trunk motion. Besides this, exercises with free weights most closely replicates the upper/lower body rotation movements. A suitable alternative represents a system consisting of an inertia measurement unit in a small box inserted on the barbell placed on the shoulders that allows evaluation of trunk rotational power in either seated or standing position. In such a case, the power is greater during standing than seated rotations of the trunk (Zemková et al., 2017b).

Typically, repetitions of a particular strength exercise with increasing weights up to the 1 RM are performed in order to obtain force-velocity or power-velocity curves. It is known that maximum force production is achieved when the movement speed is very low (Edman et al., 1976; Thorstensson et al., 1976; Binkhorst et al., 1977; Tihanyi et al., 1982; de Koning et al., 1985; De Ruiter and De Haan, 2000). As the movement speed increases, force decreases and is very low at very high speeds. Consequently, maximal values of power occurs at intermediate velocities when lifting moderate weights (i.e., 50–60% 1 RM) during typical resistance exercises such as bench presses or squats, whereas during trunk rotations it is at 30–45% 1 RM. This variation in maximal power production may be ascribed to the specificity of training adaptation.

For instance, Poór (2017) found a significant increase of mean power in the acceleration phase of trunk rotations after both the preparatory and competitive periods in tennis players at almost all weights (10–26 kg and 6–26 kg, respectively). However, its values increased significantly during trunk rotations with weights ≥12 kg in hockey players and with weights ≥10 kg in canoeists after the preparatory period only. These findings indicate that changes in trunk rotational power reflect the specificity of the training program.

Also within and between group differences in trunk rotational power and velocity may be attributed to specificity of the training involving trunk movements of different velocities under different load conditions. In particular, mean power and velocity in the acceleration phase of trunk rotation are sensitive parameters able to identify group and individual differences in athletes of various sports, such as karate, ice-hockey, tennis, golf, ballroom dancing, rock & roll dancing, judo, wrestling, canoeing, rowing, weightlifting, and bodybuilding. This parameter is also specific to asymmetric loading of core muscles during trunk rotations and may identify the likelihood of low back pain. Mean power in the acceleration phase of trunk rotations is significantly higher in the dominant than non-dominant side in golfers (11.9%) and tennis players (9.4%), whereas there are no significant side to side differences in the group of physically fit subjects (6.2%).

Taking into account the importance of core stability and strength in athlete's performance and probably also in the prediction of injuries, their assessment should be included in testing of young athletes. However, these tests involving lifting task or trunk rotations with an additional load must be performed with extreme caution. The exercises are usually performed with increasing weights up to maximal power rather than up to 1 RM. Preadolescents and adolescents should avoid using higher weights.

#### Sport-specific assessment of body balance in young athletes

Postural stability is maintained by three interrelated systems. The spinal column provides passive support, muscles give active support, and neural control centers coordinate sensory feedback from these systems. Traditionally, postural stability has been assessed under static conditions (bipedal or one-legged stance on a force plate with eyes open and eyes closed); however, these are not sensitive enough in discriminating athletes with good balance. Lower sensitivity of static posturography is a result of multiple sensory inputs involved in balance control that can compensate its small impairment. While standing on an unstable surface, this control mechanisms is taxed to a greater extent so that differences between individuals can be revealed. These conditions include a stance on a foam cushion, external perturbations generated from a platform either shifting in antero-posterior and medio-lateral direction or tilting toes up and down, and applying them directly to the body by pushing/pulling the trunk, shoulders or pelvis. For instance, subjects stand on a force plate connected to a computer with a special program that generates its movement in the horizontal plane. The protocol includes varied determinants of platform translation, such as the direction (forward, backward, left-lateral, and right-lateral), displacement (e.g., from 1 to 14 cm), and velocity (e.g., from 5 to 20 cm/s). Concurrently with measurement of center of pressure (CoP) movement, trunk movement representing roughly the center of mass (CoM) can also be monitored (Zemková et al., 2016b). Experience showed that dynamic posturography is a more sensitive and also more specific alternative for most of the athletes than systems which monitor the CoP in static conditions. Dynamic conditions can also better reveal adaptive changes in sensorimotor functions after the training (Zemková, 2010).

However, most of the dynamic posturography systems have also shortcomings. For instance, some of the platforms are insufficient to destabilize the highly skilled athletes beyond their stability limit. Others produce only unidirectional motions in the antero-posterior plane. In the case of tilting platforms, high learning effect can be observed because the subjects can predict the upcoming perturbations relatively successfully.

Another alternative is to use instrumented tests consisting of trunk repositioning and load release tasks (Reeves et al., 2007; Silfies et al., 2007). The trunk repositioning task requires the subject to passively or actively return to a neutral spine position after a predefined displacement. The load release task requires the subject to perform an isometric contraction of trunk muscles at a predefined intensity against an external load, which is thereafter released, and the trunk displacement is evaluated. Such parameters of the load release balance test measured during standing on a foam surface are able to differentiate between sedentary and physically active adults as early as from 19 years of age (Zemková et al., 2016c).

However, in many sports athletes are forced to keep balance on an unstable surface while performing tasks of various kinds simultaneously. Being able to not only stabilize and maintain balance but also to precisely and efficiently regulate positioning of the CoM may be considered as the essence of functional balance. To assess this ability, task-oriented balance tests, such as a visually-guided CoM target-matching task or a visually-guided CoM tracking task, seem to be promising. While in the first case subjects have to hit the target randomly appearing in one of the corners of the screen by horizontal shifting of CoM in an appropriate direction; in the second they have to trace, by shifting CoM, a curve flowing either in a horizontal or vertical direction. In comparison with static balance tests, task-oriented balance tests showed comparable reliability but better potential for discriminating between groups with different levels of balance capabilities. It can also more sensitively reveal the acute and long-term effect of various sensorimotor exercises on neuromuscular performance (Zemková, 2010).

A moderate correlation between parameters of these task-oriented balance tests (*r* = 0.457) and the common variance of 13% indicates that they assess distinct qualities. This is because voluntary feedback control of body position is performed under different conditions, i.e., the subject is focused either on the goal of the task (i.e., hitting the target) or on movement themselves (i.e., the positioning of the CoM). These test differences allow assessment of accuracy of regulation of body movement that requires less or more feedback processing. This is of special importance for children who regulate their CoM movement in a more conscious, effortful fashion (i.e., observed as a longer CoP trajectory) with their decisions about the action being handled in a slow, attention-demanding way (i.e., shown as a slower response time). Our experience indicates that such an assessment of balance incorporating a functional task is more suitable alternative for children and adolescents than static conditions. The accurancy of assessment of static balance can be influenced by factors, such as motivation or attentiveness, which are difficult to control in children and adolescents. Providing immediate feedback (based on visual stimuli or statoacoustic signals) may motivate young individuals to exercise as intensively as possible while reducing the level of instructor supervision. Objective feedback also allows for adjustment of the testing protocol to specific individual needs and performance capabilities. An additional benefit is that the systems may be used as the training means. One of the alternatives are computerized balance games. These are effective in speeding the learning process by enhancing the understanding of particular tasks. Indeed, Štefániková (2013) revealed that training programs consisting of visual feedback balance exercises on either a stable or an unstable surface were more effective in improvement of balance functions than exercises on unstable surfaces without visual feedback in early school age children. This novel approach is a natural step to advancing the current state of knowledge by getting objective insight into the changes in postural control system during neuromuscular training in children and adolescents.

Utilizing techniques based on motion analysis or accelerometry recordings while evaluating head, limb and trunk movements could provide additional data and complete functional diagnostics of young athletes. The use of trunk accelerometry is a cost-effective and easily applied solution for measuring body balance and human movement. In particular, the accelerometry is a valid quantitative measure of postural sway which is strongly related to task-based measures (Whitney et al., 2011). With the advent of fast wireless technology and low-cost accelerometers, their use in field-testing of various aspects of balance is now feasible.

All these techniques can be used for assessment of postural stability in sport-specific positions or after aerobic, anaerobic and resistance exercises under laboratory and sport-specific conditions in the sporting field (Zemková, 2014). A better understanding of physiological mechanisms of post-exercise balance impairment and its readjustment to baseline (Zemková and Hamar, 2014c) may serve as a basis for the design of goal-specific balance training programs to improve athletic performance and prevent a risk of injuries.

#### Sport-specific assessment of agility and speed in young athletes

The ability to perform quick movements, stop and start rapidly while focusing on an opponent or the ball plays an essential role in athlete performance. It involves perception and decision making (cognitive processing), muscle strength and change of direction speed (motor component), in addition to footwork and movement technique (technical skills).

In comparison with traditional agility tests based on pre-planned change of direction speed, novel reactive agility tests address both the cognitive (i.e., anticipation and pattern recognition) and the physical component (i.e., change of direction speed). Such testing is more sensitive in discriminating athletes of different performance levels as compared to pre-planned change of direction speed tests. For instance, Sheppard et al. (2006) discovered that the reactive agility test differentiates between Australian football players of varied performance levels, whereas sprint and sprint with change of direction tests were unable to do so. Similarly, Farrow et al. (2005) found that the highly-skilled group was faster in both the planned and reactive tests than the lesser-skilled group, whereas the moderately-skilled group was faster than the lesser-skilled group in the reactive test only. Adding reactions to given stimuli into agility tests would also reflect sport-specific situations more effectively.

Both speed of decision making and change of direction speed contribute to agility performance, although to a different extent. Agility time strongly correlates with the choice reaction time, regardless of sports specialization of athletes or their previous experience with agility training. This indicates that perception and decision making are the most influential components of agility performance. There is also a significant correlation between agility time and movement time, however only when traveling a short distance. The strength of this relationship decreases with increasing traveling distances. Greater variation in the movement time than two-choice reaction time also makes potentially meaningful differences among athletes (particularly among those of combat sports and sports games) and their differential contribution to the agility performance. For instance, cognitive and motor skills are better in karate-kumite than karate-kata competitors, when only stepping reactions are required. When moving longer distances, better agility time is in players than in goalies of soccer and ball hockey. While the motor component of agility performance seems to be predominant in players in terms of faster movement execution, in goalies it is the sensory component allowing faster decision making (Zemková, 2016b).

Hence, measurements of choice reaction time and movement time or velocity, may provide useful information on these components of agility performance in athletes with different demands on their agility skills. The contribution of movement time to the agility performance may be estimated using the Agility Index (Zemková, 2016a). It is defined as a ratio of reaction time and agility time which is divided by the previously determined coefficient for each distance traveled. This index is useful for agility testing that differs in the number of stimuli and traveling distances.

As shown, agility time significantly improved after 6-week training consisting of balance exercises performed simultaneously with reaction tasks (Zemková and Hamar, 2010). However, simple and two-choice reaction times did not change significantly. On the other hand, there was a significant increase in step initiation velocity. This faster execution of movement most likely contributed to the enhancement of agility performance. In fact, the reduction of agility time correlated significantly with an increase in step initiation velocity after the training *(r* = 0.78). Also of interest was the additional finding that the improvement in agility performance in older basketball players (on average 21 years) was greater than in their younger, less experienced counterparts (on average 15 years). This may be attributed to faster feedback control of movement execution, i.e., as experience level increased with practice, the agility time decreased.

These sports (basketball, soccer, tennis, ice hockey, badminton, racquetball, squash, volleyball, baseball, softball, lacrosse, american football, wrestling, boxing, fencing) which are ranked highest for agility require changes of movement direction while responding to stimuli, such as the ball or a player. These actions in field and court sports are performed alongside the offensive player's movements, which involves some sort of competition.

In order to mimic these sport-specific demands, the agility test should be performed under simulated competitive conditions. In such a case, agility time is significantly shorter when the test is performed by two subjects simultaneously (Agility Dual) than by one subject (Agility Single) (Zemková et al., 2013). Faster agility time recorded under simulated competitive rather than non-competitive conditions (14.3%) may be attributed to enhanced central nervous system arousal in the participants. This factor very likely contributed to a further, but not significant decrease, in agility time in the group that proceeded to the second match. Also, the learning effect may have been a factor; however this would be to a lesser extent because stimuli were randomly generated (temporally as well as spatially). The Agility Dual test should be used for testing of children and adolescents to enhance their attention and motivation. Such an exercise can also be a part of agility training in young athletes. Kováčiková (2012) found a more pronounced improvements in agility time in the Agility Dual test in the group trained under simulated competitive conditions than in the group who participated in a 8-week training without the competitive components. It seems that agility training performed for competition is a more effective method for agility skills enhancement than the training under non-competitive conditions.

This may be beneficial for children and adolescents. It is known that agility time decreases with advancing age up to maturity (Zemková and Hamar, 2014a). Its values decrease markedly from 7 to 10 years (27.1%) and from 10 to 14 years (26.5%). This is followed by a slow period from 14 to 18 years (16.5%). Participating in sport may improve their agility skills. As shown, the best agility times (<350 ms) are in athletes of racquet and combat sports with reactions to visual stimuli (table tennis, badminton, fencing, tae-kwon-do and karate), followed mainly by players of ball sports (ice-hockey, tennis, soccer, volleyball, basketball, and ball hockey with agility time of 350–400 ms), then competitors of combat sports with reactions to visual and tactile stimuli, such as aikido (400–450 ms), and finally judo and wrestling (450–500 ms; Zemková and Hamar, 2014b). In most of these sports, assessment of agility performance requires a specific approach. For this purpose, a number of test alternatives is available depending on the sport specific task (Zemková and Hamar, 2013). Experience showed that assessment of agility performance under sport-specific conditions represents a more appropriate method than the general version of the agility test.

If necessary, a visually-triggered step initiation test can be used to measure the time of foot off (onset of unloading) and foot flight time (from foot-off to foot-contact) of the first step. Alternatively, the speed of step initiation can be measured using the system based on precise analog velocity sensor. Maximal step velocity showed excellent reliability and also sufficient sensitivity to discriminate between individuals of different ages and levels of physical fitness.

In specific conditions, for instance in combat sports where upper and lower body extremities are utilized to punch and kick, the reaction time (onset of unloading in response to visual stimuli) and movement time (from foot-off to bag-contact) during the kick can be measured. The test is reliable and also able to distinguish between athletes of varied sports (tae-kwon-do and karate) and performance levels (kyu and dan) (Zagyi, 2010). The velocity of a punch in karate or box can be measured in a similar way.

The assessment of speed abilities can be completed by measuring the frequency of the movement of upper and lower limbs. The tapping of lower limbs can be performed in the standing or sitting position. The frequency of lower limb movements escalates with increasing age, the maximum recorded by adults, which then began to decline with increasing age ranges. Contact and flight times display a similar tendency, with the lowest values in subjects ranging from 19 to 24 years of age. Participating in sport may improve this ability. For instance, the best foot tapping frequency was recorded in boxers, followed by dancers, karate and taekwondo competitors. Its values were also significantly higher in karate competitors of more advanced (2. kyu to 2. Dan) than less skillful performance levels (7.−4. kyu). This test can be modified by adjusting the contact mats so that specific positions in particular sports can be simulated (e.g., fighting stance in boxing, karate or tae-kwon-do) or by increasing its duration (e.g., close to duration of performance in dancing, rock & roll or aerobics).

Additional information regarding agility and speed can be provided by using wireless timing gate systems. One has to take into account that measurement of change of direction speed may substantially differ from frequently testing straight sprinting speed by many practitioners. There is a weak relationship between change of direction speed and straight sprinting speed in highly skilled athletes. Therefore, sport-specific methods should be addressed in both the testing and training of agility skills and movement speed in young athletes.

### Gaps in current standard testing methods and proposal for future research

Analysis of the literature identified these gaps in current testing methods:

1. inadequacy in particular age periods (early childhood, late childhood, adolescence at pre-PHV, PHV, and post-PHV);
2. a low sensitivity to discriminate between young athletes of various ages and performance levels;
3. insufficent tailoring to athlete performance level (highly skilled athletes vs. their less proficient counterparts);
4. insufficent tailoring to individual needs (most of the studies were focused on groups of athletes rather than on individual's performance assessment);
5. a lack of specificity to the requirements of particular sports, very likely due to scarce information from experimental studies (most of the studies analyzed were related to sports games, namely soccer and basketball);
6. a lack of specificity to reveal the effect of training, especially resistance training (while during plyometric training, a variety of jump tests was applied, in the case of resistance training, only few studies evaluated muscle power during particular resistance exercises);
7. a limited number of variables used, very likely due to diagnostic systems being used rarely, even in recent studies.

In order to partly fill in the gap in recent studies, the Sport Longlife Diagnostic Model was proposed. Its mission is to bring personalized physical fitness testing based on user-friendly computerized systems that enable full performance assessment. It develops novel testing methodologies to address and overcome the today's available field testing. It uses innovative methods to monitor and analyse the physical fitness of individuals of different ages and performance levels in a relatively short-time period. This novel approach enables testing athlete performance under sport-specific conditions. The areas of application and the number of age and sport-specific tests are constantly growing.

The model intends to innovate the assessment of physical fitness in terms of speed, safety, precision, and functionality using new generation diagnostic equipments with the goal of making testing simpler, more effective and objective. The long-term goal is to complement current diagnostic and training methods with self-monitoring devices, virtual coaching and electronic alert in order to evaluate actual athlete performance and/or health state, and the effectiveness of physical activity interventions. The self-testing and data analysis can provide a powerful alternative to current measurement tools as well as new perspectives on applications.

A database platform for the efficient management of long-term testing is being designed. It offers exercise professionals who perform athlete's assessment an integrated application for the analysis and interpretation of subject data. The entire process of the tester-subject relationship is being managed automatically—from data collection, its analysis to the final report. Through this innovative approach, it feeds raw data into the database and fundamentally changes the way users can access, find, and match the results on populations tested. The database has an additional potential in non-sport applications such as schools, fitness and rehabilitation centers, or medical institutions.

Diagnostic centers should serve sport professionals as well as a wide variety of people who wish to protect their health through systematic innovative assessment of their physical fitness. Integrating novel systems and methods into their daily diagnostics can enhance quality, streamline the cost of testing and bring objective data to subjects allowing repeatable evaluation before, during, and after an exercise program, treatment or rehabilitation.

The Sport Longlife Diagnostic Model would represent a significant improvement over existing field testing using computerized portable equipments and applying a set of new diagnostic tools well suited for the testing of various populations. It uses sophisticated systems and methods to detect and assess the prognosis of physical fitness, and to match the subject with objective and more effective exercise testing. It provides a valid proprietary cutting-edge functional assessment platform for athletes and physically active individuals with potential for the untrained population; people with certain diseases and those recovering from injuries. End-users can be not only sport professionals and exercise practitioners but also physical education teachers, physical therapists and physicians. In this way it can be used in gyms, fitness centers and schools, as well as in rehabilitation and medical institutions.

## Author contributions

Both authors listed have made a direct and intellectual contribution to the work, and approved it for publication.

### Conflict of interest statement

The authors declare that the research was conducted in the absence of any commercial or financial relationships that could be construed as a potential conflict of interest. The reviewer HC and handling Editor declared their shared affiliation.
